# Impact of COVID-19 Pandemic on the Clinical Course and Complications of Varicella—A Retrospective Cohort Study

**DOI:** 10.3390/pediatric16020039

**Published:** 2024-06-04

**Authors:** Maja Pietrzak, Maria Pokorska-Śpiewak

**Affiliations:** 1 Pediatric Infectious Diseases Department, Regional Hospital of Infectious Diseases in Warsaw, 01-201 Warsaw, Poland; mpspiewak@zakazny.pl; 2 Department of Children’s Infectious Diseases, Medical University of Warsaw, 01-201 Warsaw, Poland

**Keywords:** varicella, chickenpox, COVID-19, migration, refugees, immunity gap

## Abstract

In this study, we aimed to characterize a cohort of children hospitalized due to varicella before and after the outbreak of the COVID-19 pandemic. Medical charts of all children hospitalized in the Regional Hospital of Infectious Diseases in Warsaw due to varicella in the years 2019 and 2022 were retrospectively analyzed and compared. In total, 221 children were included in the analysis; 59 of them were hospitalized in 2019, whereas 162 were hospitalized in 2022. Children hospitalized in 2022 were older than those reported in 2019 (median 4.0 vs. 3.0 years, *p* = 0.02). None of the hospitalized children received complete varicella vaccination. The most common complication in both years was bacterial superinfection of skin lesions, found in 156/221 (70.6%) of patients. This complication rate was higher in 2022 (50.8% in 2019 vs. 77.8% in 2022, *p* = 0.0001), OR = 3.38, 95% CI: 1.80–6.35. Moreover, skin infections in 2022 more often manifested with cellulitis (in 2022 13.6% vs. 3.4% in 2019, *p* = 0.03), OR = 4.40, 95% CI: 1.00–19.33. Sepsis as a complication of varicella was almost five-fold more prevalent in 2022 than in 2019 (*p* = 0.009), OR = 5.70, 95% CI: 1.31–24.77. Antibiotic use increased between 2019 and 2022 (71.2% vs. 85.2%, *p* = 0.01). Furthermore, patients were treated more frequently with the combination of two different antibiotics simultaneously (only 3.4% of patients in 2019 compared to 15.4% in 2022, *p* = 0.01). Primary infections with varicella zoster virus in 2022 led to a more severe course of the disease.

## 1. Introduction

Varicella-zoster virus (VZV) spreads through the airborne route and is highly contagious; around 90% of susceptible people exposed in household settings will develop the infection. The primary infection manifests as varicella and in susceptible (unvaccinated) populations, infection usually occurs in early childhood, with an annual rate of around birth cohort [1]. Despite a usually mild course in immunocompetent children, varicella presents a crucial risk to immunocompromised people and pregnant women. For this reason, the implementation of the varicella vaccine in 1995 was a major breakthrough. The use of varicella vaccine for over 20 years has proven its efficacy and safety. Countries that implemented it into universal immunization programs have observed lower rates of complications, hospitalizations, and deaths. It has also been proven to be cost-effective [2].

Although some European countries implemented mandatory national vaccination against varicella, in many others, varicella vaccination remains recommended for specific risk groups and therefore the vaccine coverage remains low [3,4]. In Poland, varicella immunization is recommended and obligatory only for children from risk groups (with immunodeficiencies, before immunosuppressive treatment and chemotherapy, children institutionalized in orphanages and nursing and care facilities, etc.) [5]. Due to the control measures and population containment implemented during the epidemic of coronavirus disease 2019 (COVID-19), a greater number of varicella-susceptible children were to be expected in 2022. Those children would also be older, placing more of them in the age-related risk group for more severe varicella (usually children older than 12 years old are considered as higher risk) [6]. Moreover, we presumed that more children were at risk of acquiring two different infectious diseases due to the observed immunity gap, thus risking a graver progression of the illness [7,8].

The aim of this study was to characterize the epidemiology, clinical course, and complications of varicella in the cohort of hospitalized children before the onset of COVID-19 and after almost two years of lockdown due to the pandemic. In addition, in 2022, Poland experienced a huge migration wave from Ukraine after the start of the Russian Invasion of Ukraine in February 2022. We compared the changes in demographics, duration of symptoms, complications, and treatment of patients hospitalized in 2019 and 2022.

## 2. Materials and Methods

Medical charts of all children hospitalized in the Regional Hospital of Infectious Diseases in Warsaw, Poland, due to varicella in the year 2019 and the year 2022 were retrospectively analyzed. Department of Children’s Infectious Diseases is a tertiary healthcare department dedicated to children with contagious diseases. We have chosen the year 2019 as the representative year from the pre-pandemic period because, according to the data from the National Institute of Health, the incidence rate was stable in the last few years preceding the pandemic [9]. The year 2022 was the first year after the beginning of the pandemic in which schools and nurseries in Poland were fully open with little or no containment measures. Inclusion criteria were age 0–18 years and a final diagnosis of varicella. Age, sex, the month of the onset of symptoms, history of varicella vaccination, immunosuppression, comorbidities, clinical course, complications, and treatment were analyzed in both groups. Varicella was diagnosed based on typical clinical presentation irrespective of the confirmed contact with a person with diagnosed chickenpox. Sepsis and other complications were diagnosed clinically or combined with the laboratory findings. Infant was defined as a child under 1 year of age. A refugee was defined as a person who, due to external aggression in his country of origin, fled the country. The median time of lesions was defined as the time till the crusting of all lesions. The median time of fever was time since onset of temperature > 38.0 degrees Celsius till normalization below this setpoint. The median time of hospitalization was time in days spent in hospital.

Statistical analysis was performed using MedCalc Statistical Software version 22.007 (MedCalc, Ostend, Belgium, https://www.medcalc.org, accessed on 20 May 2024). Continuous variables were presented as the medians with interquartile ranges (IQRs) and were compared using the Mann–Whitney U test.

Categorical variables were presented as numbers with percentages and were compared using the χ^2^ test. A two-sided *p*-value of <0.05 was considered significant.

## 3. Results

### 3.1. Study Group

We included 221 patients diagnosed with varicella: 59 hospitalized in 2019 and 162 hospitalized in 2022. The baseline demographic and epidemiologic characteristics of the study group are shown in Table 1.

**Figure 1:**
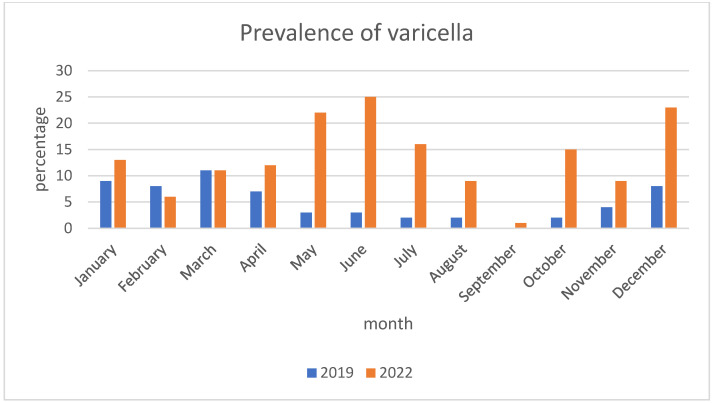
Monthly distribution of varicella.

Children hospitalized in 2022 were older than those reported in 2019 (median 4.0 vs. 3.0 years, *p* = 0.02), and the proportion of infants was lower (14.8% vs. 27.1%, *p* = 0.03). The proportion of male and female patients was equal. The numbers of patients diagnosed in the subsequent months in both years are presented in Figure 1. We found a significant difference (*p* = 0.0011) in the seasonal distribution (winter-spring vs. summer-fall) between the two observed years, with unusually high prevalence in the late spring and summer months of 2022. In 2019, 98.3% of hospitalized patients were not vaccinated against varicella, and in 2022, 99.4% were not vaccinated, which shows no difference. None of the hospitalized children received full (two doses) varicella vaccination. We found a significant difference between sources of infection in 2019 and 2022 (*p* = 0.007), with more cases of unknown contact and surprisingly fewer from refugee camps in 2022. Household contact constituted 39% of cases in 2019 and 34.6% in 2022, daycare/school contact was 32.2% and 33.3%, respectively, refugee camps 10.2% and 1.3%, and the origin remained unknown in 18.6% and 30.9%. Forty-eight out of 221 patients (21.7%) suffered from comorbidities—the most common was acute gastroenteritis (5.4%), followed by influenza (3.6%), RSV infection (1.8%), eczema (1.8%), and UTI (1.8%). Patients with immunosuppression constituted only 2.3% of all patients.

### 3.2. Clinical Presentation

The clinical presentation of varicella in the analyzed period is shown in Table 2. In 2022, we observed more severe cases—more patients were admitted in a serious general condition, fever lasted longer (4 days vs. 2, *p* < 0.0001), and hospitalization was prolonged (5 vs. 4 days, *p* = 0.01). The most common complication and cause of admission in both years was bacterial superinfection of skin lesions, which was found in 156/221 (70.6%) of patients, but the rate of this complication was significantly higher in 2022 (50.8% in 2019 vs. 77.8% in 2022, *p* = 0.0001), OR = 3.38, 95% CI: 1.80–6.35. The etiology of the skin superinfection was more often detected in 2022 compared to 2019, OR = 9.07, 95% CI: 2.71–30.4. The most common causative agents were found to be *Streptococcus pyogenes* and *Staphylococcus aureus*, which were detected in the cultures of skin swab samples more often in the year 2022 (5.1% in 2019 versus 16.0% in 2022, *p* = 0.03 for *S. pyogenes* and, respectively, 1.7% versus 16.0%, *p* = 0.004 for *S. aureus*). Scarlet fever was slightly more common in 2022, OR = 1.11, 95% CI: 0.50–2.44. Moreover, skin infections in 2022 more often manifested in cellulitis (in 2019 3.4% versus in 2022 13.6%, *p* = 0.03) with OR = 4.40, 95% CI: 1.00–19.33. In 2022, sepsis as a complication of varicella was almost five-fold more prevalent than in 2019 (*p* = 0.009) with OR = 5.70, 95% CI: 1.31–24.77. On the other hand, we observed fewer neurological complications (*p* = 0.01) with OR = 0.65, 95% CI 0.44–0.94, especially seizures (*p* = 0.006) in 2022. Other most common complications of varicella infection included otitis media found in 19/221 (8.6%) of patients, pneumonia in 16/221 (7.2%), and syncope in 7/221 (3.2%). We observed eye involvement in 84/221 (38%) patients but with no lesions found in the cornea. As shown in Table 3, no significant difference in the levels of inflammation markers was found.

### 3.3. Treatment

Medications used in studied patients are shown in Table 4. Due to the prevalence of bacterial complications of varicella, most of the patients required antibiotic therapy. A major rise in antibiotic use was seen between 2019 and 2022 (71.2% versus 85.2%, *p* = 0.01). Furthermore, patients were treated more frequently with the combination of two different antibiotics simultaneously (only 3.4% of patients in 2019 compared to 15.4% in 2022, *p* = 0.01). Over 60% of hospitalized patients received acyclovir. In 2022, acyclovir was more commonly administered orally (*p* = 0.01). We also noticed an increase in the use of antipyretics (62.7% versus 85.2%, *p* = 0.0003) and intravenous fluids (76.3% versus 88.9%, *p* = 0.01) in the year 2022.

## 4. Discussion

In this retrospective study, we characterized a group of 221 children hospitalized due to varicella in two periods: the years 2019 and 2022. Thus, it was possible to compare the epidemiological and clinical presentation of the disease one year before the declaration of the COVID-19 pandemic by the World Health Organization (WHO) in March 2020 and after two years of the pandemic, in the period of lesser precaution measures with fully opened institutions like schools and kindergartens as well as entertainment areas such as playgrounds. A major rise in the number of varicella cases requiring hospitalization was observed. One of the possible explanations for this tendency might be a general surge in the prevalence of varicella. In populations that have experienced strict COVID-19 precaution measures, compensatory outbreaks of several infectious diseases have been reported.

For example, in the fall/winter season of 2022/2023, we experienced, similarly to other European countries, a wave of respiratory syncytial virus (RSV) infections. This wave started much earlier, the hospitalization rate was high and affected not only infants but also children of older age groups who were not previously exposed to the virus due to the lockdown [10].

Yearly variation might have contributed as well due to varicella’s interepidemic cycle of 2–5 years [11]. However, according to the epidemiological reports of the National Institute of Public Health—National Institute of Hygiene, in 2019, over 180,000 cases of varicella were diagnosed in Poland, of which 1156 patients were hospitalized [12]. In comparison, in the year 2022, 171,000 cases of varicella were reported, with 839 patients requiring hospitalization [13]. Therefore, we cannot state that the observed higher number of children hospitalized due to varicella in 2022 was caused by the general increase in overall cases of varicella in Poland. We also did not observe a significant rise in the prevalence of varicella in the period of the massive immigration of children from Ukraine (over 1 million) after the onset of the Russian Invasion of Ukraine (March–April 2022), nor did refugees constitute a significant proportion of patients [14]. What we think might be an accurate explanation for the observed trend is that in 2022, in general, we observed a greater number of infections in children with more severe clinical courses. What is more, Warsaw (Poland), where our hospital is located, provided a home for almost 90,000 war refugees aged 0–19 till the end of May 2022 [15]. The general pediatric hospitals might have had difficulties providing isolation for varicella cases and redirected more patients to our hospital.

We observed a statistically significant change in the age distribution of patients, with a greater proportion of older children and fewer infants. This may be due to a period of COVID-19 isolation during which some children missed the typical age of acquiring varicella. Presumably due to the constantly decreasing number of births in Poland, we are observing a lesser proportion of infants in general and thus a lesser proportion of infants with varicella who require hospitalization.

According to well-established knowledge, varicella peaks in winter and spring [16]. In 2019, the distribution of varicella hospitalization rate was consistent with the above. In May, June, and July of 2022, an unusually high number of cases was observed—possibly a late surge after the immigration wave.

Due to the retrospective character of the study, we could not assess the direct impact of COVID-19 on the morbidity and rate of complications. Yet, according to Naeimi et al., by the end of 2021 around 30–50% of children worldwide have had COVID-19 [17]. In the study based in Poland, the seroprevalence of SARS-CoV-2 antibodies was assessed at the level of 57% [18]. We can assume with a quite high degree of certainty that a greater number of our patients had suffered from COVID-19 in the two years preceding the hospitalization.

In Poland, among many other European countries, national vaccination against varicella has not been implemented. According to the WHO, countries are encouraged to introduce routine varicella immunization if they can reach and sustain vaccine coverage of at least 80%. This has been found to greatly reduce disease and economic burden and prevent shifting varicella infection to older age groups [16]. In our study, none of the hospitalized children received full varicella vaccination. In general, 99.1% of studied patients did not receive any dose of the vaccine. This finding corresponds with the vaccine efficacy estimated between 98% for two doses and 94% for one dose [19]. In the absence of a universal varicella vaccine, it has been estimated that Poland spends a mean value of EUR 22.8 million annually for varicella, placing 6th among European countries in terms of indirect and direct costs [20].

Immunosuppression among our patients did not account for a large number of cases and did not differ significantly between the two comparative years.

As for the source of infection, which remains crucial from an epidemiological perspective, it was established for 83.4% of cases in 2019 and for 69.1% in 2022 (*p* = 0.007). Household and daycare contacts constituted over two-thirds of sources and as such could be easily preventable with the use of varicella vaccine as pre- and postexposure prophylaxis. Knowledge about the source of infection remains crucial when deciding upon the administration of acyclovir, for which household contact remains an indication [6].

When discussing the clinical course of the disease, in 2022 we observed a significantly longer duration of both fever and hospitalization, which correlates with the observed higher number of cases with a severe course of the illness. In one of the studies conducted in France, the most common varicella complications included cutaneous (34%), digestive (19.8%), respiratory (17.6%), neurological (15.3%), and ear, nose, and throat (ENT) complications (8.8%) [21]. In another French multicenter study, skin infections were also the main complication of varicella, constituting up to 47% of all complications [22]. We also observed that bacterial superinfections of the skin were the most common varicella complication overall, with a significant 1.5-fold (*p* = 0.0001) increase in 2022. The main causative pathogens of this complication were *S. aureus* and *S. pyogenes*, which were responsible for up to 29% and 27% of skin infections, respectively. In the last year across Europe, an increase in group A streptococcal (GAS) infections has been observed. In one of the studies performed in the Netherlands, there was a sevenfold rise in the number of invasive GAS infections compared to years before the COVID-19 pandemic [23]. In our study, we also observed a threefold increase in infections triggered by *S. pyogenes* and over ninefold in infections caused by *S. aureus*. In almost every fifth patient with skin complications, scarlet fever was diagnosed. In the year 2022, we observed more severe skin complications. There was a fourfold increase in cellulitis (*p* = 0.03) and an almost fivefold (*p* = 0.009) increase in the incidence of sepsis. Although the global increase in streptococcal infections played a huge role in the observed rise in skin complications, we also observed a rise in skin complications caused by *S. aureus*. One of the possible explanations is the higher use of telemedicine. Before the COVID-19 pandemic, telemedicine (mainly phone calls) was not used in pediatrics in Poland. However, that has changed during the pandemic and afterwards and might have led to the late recognition of skin complications and therefore late treatment and higher admission rates. Other main complications observed in our study group were otitis media, pneumonia, syncope, cerebellitis, and arthritis (see Table 2). Although basic laboratory inflammation markers did not differ significantly between the comparative years, in 2022 we saw greater use of antibiotics in general, and more often patients required two antibiotics given simultaneously, which corresponds with the observed trend of more severe skin complications followed by cellulitis and sepsis. The most commonly used combination of antibiotics was III-generation cephalosporin (due to often high inflammation markers and the possibility of sepsis) plus clindamycin. Adjunctive use of clindamycin has been proven to improve outcomes in patients with invasive GAS infections due to inhibition of the production of bacterial proteins and superantigens [24,25].

In accordance with longer hospitalizations, longer duration of fever, and a greater number of sepsis, we observed higher use of antipyretics and intravenous fluids.

This study has limitations. It has been conducted retrospectively and thus did not permit us to distinguish between causes and effects. In addition, our study was conducted in a hospital setting, which limited the cross-section of the patient population, representing more severely ill children. Due to the lack of a Pediatric Intensive Care Unit (PICU) in our hospital, patients with critical status or potentially lethal neurological complications, such as stroke or encephalitis, were not admitted or were transferred to the hospitals with the PICU/neurology department. Some of the variables, e.g., patient’s general condition at admission were assessed arbitrarily and might have depended on the experience of the admitting doctor.

To the best of our knowledge, we present the only study documenting differences in the epidemiology and clinical presentation of varicella before the onset of the COVID-19 pandemic and after. In addition, our study reflects changes in the epidemiology of varicella not only in the context of the world pandemic but also in the period of the largest migration crisis in Europe since World War II. Given the more than 20-year observance period of the varicella vaccine, we can strongly recommend obligatory national varicella immunization for children. The vaccine has been proven to be safe and initial doubts about long-term efficacy, varicella outbreaks in the older population, or risk of earlier onset of shingles have been dispelled.

## 5. Conclusions

In conclusion, primary infections with varicella zoster virus in 2022 led to a more severe course of the disease, with higher rates of skin complications and sepsis compared to the pre-pandemic year 2019. Thus, further studies are needed to analyze the tendency of varicella infections, rates of complications, and precautions needed to ensure the best management of VZV infections, especially implementing mandatory varicella vaccination in the national immunization program.

## Figures and Tables

**Table 1:** Baseline Demographic and Epidemiologic Characteristics of the Study Group.

			Total *n* = 221	2019 *n* = 59	2022 *n* = 162	*p* Value (2019 vs. 2022)
Sex, *n* (%)		Male	112 (51)	29 (49)	83 (51)	0.78
		Female	109 (49)	30 (51)	79 (49)	
Median age in years (IQR)			3.5 (1.0; 6.0)	3.0 (0.5; 5.4)	4.0 (1.0–6.0)	0.02
Infants, *n* (%)			40 (18)	16 (27.1)	24(14.8)	0.03
Ukrainian refugee, *n* (%)			8 (3.6)	0	8 (4.9)	0.08
Month of infection, *n* (%)	Winter-spring (December–May)		133 (60.2)	46 (78)	87 (53.7)	0.0011
	Summer-fall (June–November)		88 (39.8)	13 (22)	75 (46.3)	
Coronavirus disease 2019, *n* (%)	Vaccinated		Not applicable		2 (1.2)	Not applicable
	Not vaccinated				64 (39.5)	
	Not qualified for vaccination				96 (59.3)	
Varicella immunization, *n* (%)	Not vaccinated		219 (99.1)	58 (98.3)	161 (99.4)	0.45
	1 dose/postexposure		2 (0.9)	1 (1.7)	1 (0.6)	
	Fully vaccinated		0	0	0	
Immunosuppression, *n* (%)			5 (2.3)	2 (3.4)	3 (1.9)	0.49
Source of infection, *n* (%)	Unknown		61 (27.6)	11 (18.6)	50 (30.9)	0.007
	Household		79 (35.7)	23 (39)	56 (34.6)	
	Daycare/Schools.py		73 (33)	19 (32.2)	54 (33.3)	
	Refugee camp		8 (3.6)	6 (10.2)	2 (1.3)	
Comorbidities, *n* (%)			48 (21.7)	14 (23.7)	34 (21)	0.66

**Table 2:** Comparison of clinical presentation of varicella in children hospitalized in 2019 and 2022.

		Total (*n* = 221)	2019 (*n* = 59)	2022 (*n* = 162)	*p* Value (2019 vs. 2022)
General condition, *n* (%)	Good	55 (24.9)	15 (25.4)	40 (24.7)	0.52
	Fair	138 (62.4)	39 (66.1)	99 (61.1)	
	Serious	28 (12.7)	5 (8.5)	23 (14.2)	
Median Time of (in days), IQR	Lesions	6 (5–8)	6 (5–7)	7 (5–8)	0.17
	Fever	3 (1–4)	2 (0–3)	4 (2–5)	<0.0001
	hospitalization	4 (4–6)	4 (3–5)	5 (4–7)	0.01
Skin complications, *n* (%)		159 (71.9)	31 (52.5)	129 (79.6)	0.0001
Bacterial superinfection, *n* (%)		156 (70.6)	30 (50.8)	126 (77.8)	0.0001
Etiology, *n* (%)	*S. pyogenes*	29 (13.1)	3 (5.1)	26 (16.0)	0.03
	*S. aureus*	27 (12.2)	1 (1.7)	26 (16.0)	0.004
	*S. pyogenes + S. aureus*	3 (1.4)	0	3 (1.9)	0.29
Scarlet fever, *n* (%)		40 (18.1)	10 (16.9)	30 (18.5)	0.79
Cellulitis, *n* (%)		24 (10.9)	2 (3.4)	22 (13.6)	0.03
Sepsis, *n* (%)		29 (13.1)	2 (3.4)	27 (16.7)	0.009
Neurological complications *n* (%)	Total	12 (5.4)	5 (8.5)	7 (4.3)	0.01
	Meningitis	0	0	0	-
	Cerebellitis	6 (2.7)	3 (5.1)	3 (1.9)	0.19
	encephalitis	1 (0.5)	0	1 (0.6)	0.54
	Seizures	5 (2.3)	4 (6.8)	1 (0.6)	0.006
Eye involvement, *n* (%)		84 (38)	21 (35.6)	63(38.9)	0.65
Other complications, *n* (%)	Total	58 (26.2)	21 (35.6)	37 (22.8)	0.05
	syncope/unconsciousness	7 (3.2)	2 (3.4)	5 (3.1)	0.9
	otitis media	19 (8.6)	7 (11.9)	12 (7.4)	0.29
	Pneumonia	16(7.2)	5 (8.5)	11 (6.8)	0.66
	Other	13 (5.8)	6 (1.0)	7 (4.3)	0.02

**Table 3:** Comparison of inflammation markers in patients hospitalized for varicella.

	Total	2019	2022	*p* Value (2019 vs. 2022)
Leukocytes Median (IQR)	8.8 (6.7–12.3)	9.5 (7.4–13.6)	8.5 (6.5–11.9)	0.13
CRP Median (IQR)	14.0 (6.3–28.8)	9.5 (6.0–30.0)	16 (7.0–28.3)	0.27
PCT Median (IQR)	0.29 (0.16–0.88)	0.26 (0.16–1.24)	0.31 (0.16–0.87)	0.79

**Table 4:** Treatment of varicella and its complications.

		Total (*n* = 221)	2019 (*n* = 59)	2022 (*n* = 162)	*p* Value (2019 vs. 2022)
Number of antibiotics, *n* (%)	None	41 (18.6)	17 (28.8)	24 (14.8)	0.01
	One	150 (67.9)	40 (67.8)	110 (67.9)	0.98
	Two	27 (12.2)	2 (3.4)	25 (15.4)	0.01
	Three	3 (1.3)	0	3 (1.9)	0.29
Route of administration, *n* (%),	Oral	20	7	13	0.19
	Intravenous	27	8	19	0.24
	Sequential	133	27	106	0.10
Acyclovir, *n* (%)	Total	133 (60.1)	33 (55.9)	100 (61.7)	0.43
	Oral	116 (52.5)	24 (40.7)	92 (56.8)	0.01
	Intravenous	10 (4.5)	5 (8.5)	5 (3.1)	
	Sequential	7 (3.2)	4 (6.8)	3 (1.9)	
Antihistamines, *n* (%)		126 (57.0)	28 (47.5)	98 (60.5)	0.08
Antipyretics, *n* (%)		175 (79.2)	37 (62.7)	138 (85.2)	0.0003
Intravenous fluids, *n* (%)		189 (85.5)	45 (76.3)	144 (88.9)	0.01
Mannitol, *n* (%)		8 (3.6)	3 (5.1)	5 (3.1)	0.48
Steroid, *n* (%)		10 (4.5)	3 (5.1)	7 (4.3)	0.80

## Data Availability

The data presented in this study are available on request from the corresponding author. The data are not publicly available, because the study based on data collected in the Department’s database.
